# Reduction of acute mild stress corticosterone response and changes in stress‐responsive gene expression in male Balb/c mice after repeated administration of a *Rhodiola rosea* L. root extract

**DOI:** 10.1002/fsn3.1249

**Published:** 2019-10-22

**Authors:** Anne‐Laure Dinel, Isabelle Guinobert, Céline Lucas, Claude Blondeau, Valérie Bardot, Isabelle Ripoche, Lucile Berthomier, Véronique Pallet, Sophie Layé, Corinne Joffre

**Affiliations:** ^1^ Integrated Nutrition and Neurobiology, UMR 1286 INRA Bordeaux France; ^2^ Integrated Nutrition and Neurobiology, UMR 1286 Bordeaux University Bordeaux France; ^3^ Integrated Nutrition and Neurobiology, UMR 1286 NutriBrain Research and Technology Transfer Bordeaux France; ^4^ Groupe Pileje Paris France; ^5^ Naturopôle, Les Tiolans Saint‐Bonnet de Rochefort France; ^6^ CNRS, SIGMA Clermont Clermont‐Ferrand Chemistry Institute, Clermont Auvergne University Clermont Ferrand France

**Keywords:** acute mild stress, circadian rhythm, corticosterone, nutritional supplementation, rhodiola

## Abstract

*Rhodiola rosea* L. (*R. rosea*) is an adaptogenic plant increasing body resistance to stress. Its efficacy has been evidenced mainly in chronic stress models, data concerning its effect in acute stress and underlying mechanisms being scarce. The objective was to investigate the effect of repeated doses of a *R. rosea* hydroethanolic root extract (HRE) on hypothalamic pituitary adrenal response in a murine model of acute mild stress and also the mechanisms involved. Stress response was measured in Balb/c mice having received by gavage HRE (5 g/kg) or vehicle daily for 2 weeks before being submitted to an acute mild stress protocol (open‐field test then elevated plus maze). Corticosterone was measured in plasma from mandibular vein blood drawn before and 30, 60, and 90 min after initiation of the stress protocol. Mice were sacrificed at 90 min, and the hippocampus, prefrontal cortex, and amygdala were excised for high‐frequency RT‐PCR gene expression analysis. At 30 min after acute mild stress induction, corticosterone level in mice having received the HRE was lower than in control mice and comparable to that in nonstressed mice in the HRE group. HRE administration induced brain structure‐dependent changes in expression of several stress‐responsive genes implicated in neuronal structure, HPA axis activation, and circadian rhythm. In the acute mild stress model used, *R. rosea* HRE decreased corticosterone level and increased expression of stress‐responsive genes, especially in the hippocampus and prefrontal cortex. These findings suggest that *R. rosea* HRE could be of value for modulating reactivity to acute mild stress.

## 1 INTRODUCTION

Stress is the physiological reaction to environmental threats or pressure and can be self‐driven or of external origin (Anghelescu, Edwards, Seifritz, & Kasper, 2018). It is manifested by a wide variety of physical and psychological symptoms. If persistent and left untreated, stress can result in serious health problems including burnout, depression, post‐traumatic stress disorder, anxiety, and cardiovascular, gastrointestinal, neurological, and musculoskeletal diseases. Stress appears to be a particular problem in our modern society. Work‐related stress is experienced by all sections of society, being estimated to affect 22% of the European workforce (Milczarek & Gonzales, 2009). The World Health Organization has called stress “the health epidemic of the 21st century,” recognizing its substantial impact on personal life and also its social and economic consequences (Anghelescu et al., 2018; Subhani et al., 2018).

Stress management strategies include nonpharmacological approaches, such as cognitive behavioral therapy and relaxation, but recourse to pharmacological treatment is standard if stress and its symptoms become harmful. Anxiolytics and antidepressants, associated with known risks of adverse effects and dependency, are generally indicated for more severe situations. Several plants, including chamomile, melissa, and rhodiola, have been shown to be valuable for managing stress and its consequences, with fewer adverse effects and a lower risk of dependency (Sarris, McIntyre, & Camfield, 2013). *Rhodiola rosea* L. (rosenroot or golden root), manifesting adaptogenic properties, is among those most widely used (Anghelescu et al., 2018; Kasper & Dienel, 2017). Extracts of adaptogenic plants can normalize body functions and reinforce systems compromised by stress (Anghelescu et al., 2018). They have no specific pharmacological properties and act by increasing resistance to a broad spectrum of adverse expressions of stress. Preclinical in vivo and ex vivo studies in animal models and experiments on cell lines have highlighted several biochemical and pharmacological stress‐reducing properties of *R. rosea* extracts (Abidov, Crendal, Grachev, Seifulla, & Ziegenfuss, 2003; Olsson, von Scheele, & Panossian, 2009; Panossian, Hambardzumyan, Hovhanissyan, & Wikman, 2007; Panossian, Hovhannisyan, Abrahamyan, Gabrielyan, & Wikman, 2009). In clinical studies, various extracts of *R. rosea* were found to be effective and safe, improving mental work capacity, concentration, task performance, fatigue, burnout symptoms, and overall mood, besides reducing stress level and self‐reported mild anxiety (Cropley, Banks, & Boyle, 2015; Darbinyan et al., 2000; Edwards, Heufelder, & Zimmermann, 2012; Kasper & Dienel, 2017; Panossian, Wikman, Kaur, & Asea, 2009; Punja, Shamseer, Olson, & Vohra, 2014). *R. rosea* was approved by the European Medicines Agency Committee on Herbal Medicinal Products for the indication “temporary relief of symptoms of stress such as fatigue and sensation of weakness” (EMA/HPMC, 2012).

Stress response typically begins with activation of the hypothalamus–pituitary–adrenal (HPA) axis, one of the main stress response pathways, and the production of corticosteroids (Anghelescu et al., 2018; Subhani et al., 2018). Acute or chronic stress produces characteristic changes in the HPA axis, including an increase in cortisol in humans and corticosterone in rodents, as well as a reduction in the sensitivity of the HPA axis to feedback down‐regulation (Anghelescu et al., 2018; Panossian, Wikman, et al., 2009). Chronic stress results in persistent elevation of cortisol or corticosterone levels, which may lead to fatigue, depression, and other symptoms (Anghelescu et al., 2018). The reduction in stress‐induced damage by *R. rosea* is characterized by a decrease in or the prevention of hormonal changes characteristic of stress, including cortisol or corticosterone release, as shown in humans suffering from chronic stress following administration of the standardized *R. rosea* root extract SHR‐5 during 28 days (Olsson et al., 2009) and in rabbits subjected to acute stress after 7 days of SHR‐5 administration (Panossian et al., 2007). HPA axis modulation by *R. rosea* extracts also involves the inhibition of stress‐induced protein kinases and nitric oxide in animals (Panossian, Wikman, et al., 2009). The HPA axis is not the only target of *R. rosea*. For instance, *R. rosea* extracts stimulated energy metabolism in rodents via the activation of ATP synthesis in mitochondria (Abidov et al., 2003) and might protect against neurodegenerative brain diseases through antioxidative and anti‐inflammatory mechanisms (Lee et al., 2013; Zhang, Zhu, Jin, Yan, & Chen, 2006).

Investigations of the molecular mechanisms underlying central corticosteroid action following a stress event led to the identification of genetic pathways and, in particular, stress‐responsive genes (Hunter et al., 2016; Kohrt et al., 2016). Modification of target gene transcription, the so‐called genomic action of corticosteroids, is therefore most likely one of the main mechanisms underlying corticosteroid action in the brain (Gray, Kogan, Marrocco, & McEwen, 2017). These genomic effects can occur within 15–30 min after the activation of corticosteroid receptors and may last for less than an hour or up to several days, depending on the duration of exposure to the hormone and the type of stress (Dong, Poellinger, Gustafsson, & Okret, 1988; Morsink, Joels, et al., 2006). These stress‐responsive genes are divided into several functional classes according to their implication in energy metabolism, signal transduction, neuronal structure, vesicle dynamics, neurotransmitter catabolism or cell adhesion, their encoding of neurotrophic factors and their receptors, and their involvement in the regulation of glucocorticoid signaling (Andrus et al., 2012; Datson, Morsink, Meijer, & de Kloet, 2008; Datson et al., 2012; Hunter et al., 2016). The effects of *R. rosea* extracts on these stress‐responsive genes are unknown. Furthermore, all the data on *R. rosea* reported so far have been obtained following intense stress, either acute or chronic. Characterizing the effects of *R. rosea* on the HPA axis and stress‐responsive gene transcription under acute mild stress conditions would contribute to a better understanding of how extracts of this adaptogenic plant act to prevent the negative effects of stress.

The purpose of this study was therefore to evaluate, in a murine model of acute mild stress, the effects on the HPA axis of repeated administration of a hydroethanolic root extract (HRE) of *R. rosea*, phytochemically characterized by high‐performance thin‐layer chromatography (HPTLC) and ultra‐high‐performance liquid chromatography coupled with mass spectrometry (UHPLC‐MS). Corticosterone secretion and stress‐responsive gene expression were determined in the prefrontal cortex (PFC), amygdala, and hippocampus, the main structures implicated in stress management.

## 2 MATERIAL AND METHODS

### 2.1 Preparation of the *R. rosea *HRE

The *R. rosea* HRE was obtained according to the patented process WO2001056584A1 by crushing frozen fresh roots of *R. rosea* and leaching with 20%–70% (v/v) ethanol. The extract was then concentrated under reduced pressure to evaporate ethanol. The salidroside titer was adjusted within the range of 0.7–1.4 mg/ml by adding glycerin to the concentrated extract. The batch of HRE used in this study (16H321), containing 83% glycerin, had a salidroside content of 1.02 mg/ml and a dry drug: dry genuine extract ratio of 17:1. This glycerin‐containing HRE corresponds to the standardized extract of *R. rosea* marketed in France under the brand name “Extrait de plante fraîche standardisé (EPS) *R. rosea”* (PiLeJe Laboratoire, France).

### 2.2 LC/MS analysis of the *R. rosea* HRE

UHPLC analysis was performed on an Ultimate 3000 RSLC UHPLC system (Thermo Fisher Scientific Inc., MA, USA) coupled to a quaternary rapid separation pump (Ultimate autosampler) and a rapid separation diode array detector. Compounds were separated on an Uptisphere Strategy C18 column (25 × 4.6 mm; 5 μm; Interchim, Montluçon, France), maintained at 40°C. The mobile phase was a mixture of 0.1% (v/v) formic acid in water (phase A) and 0.1% (v/v) formic acid in acetonitrile (phase B). The gradient of phase A was 100% (0 min), 80% (10 min), 73% (35 min), 0% (40–50 min), and 100% (51–60 min). The flow rate was 0.8 ml/min and the injection volume 10 µl. The UHPLC system was connected to an Orbitrap mass spectrometer (Thermo Fisher Scientific Inc., MA, USA) operating in negative electrospray ionization mode. Source operating conditions were as follows: 3 kV spray voltage for negative mode; 320°C heated capillary temperature; 400°C auxiliary gas temperature; sheath, sweep, and auxiliary gas (nitrogen) flow rate 60, 17.5, and 3.5 arbitrary units, respectively; and collision cell voltage between 20 and 50 eV. Full scan data were obtained at a resolution of 35,000 whereas MS^2^ data were obtained at a resolution of 17,500. Data were processed using Xcalibur software (Thermo Fisher Scientific Inc., MA, USA). The constituents of the *R. rosea* HRE were identified according to their retention times and mass spectral data and by comparison with authentic standards, if available, or otherwise with published data.

### 2.3 HPTLC analysis of *R. rosea* HRE

Standards were diluted in methanol at a concentration of 0.5 mg/ml for rosavin and 0.1 mg/ml for salidroside (Sigma Aldrich, Saint Louis, USA). One mL of the *R. rosea* HRE (without added glycerol) was diluted in 3 ml of a mixture of 50% ethanol and water (50/50: v/v). The resultant solution was shaken and centrifuged for 3 min at 6,600 g. The supernatant solution was transferred into individual vials and then analyzed by HPTLC. HPTLC analysis was performed on 200 × 100 mm silica gel 60 F 254 HPTLC glass plates (Merck, Darmstadt, Germany), using a Camag HPTLC system (Muttenz, Switzerland) equipped with an Automatic TLC Sampler (ATS 4), an Automatic Developing Chamber ADC2 with humidity control, a TLC Visualizer, WinCATS software and for derivatization, a Chromatogram Immersion Device III, and a TLC Plate Heater III. Standard solutions and samples were applied as bands 8.0 mm wide, up to a 8.0 mm from the lower edge of the plate and 15 mm from the left and right edges. The space between bands was 11.3 mm, and each plate contained 16 tracks. The development distance was 70.0 mm from the lower edge of the plate. The temperature within the developing chamber was set at 21°C and the relative humidity at 37%. The mobile phase was a solution of ethyl acetate, water, formic acid, and methanol (volume ratio: 77/10/2/13). Derivatization was performed by dipping (speed: 5, time: 0) in a reagent comprising 10% sulfuric acid in methanol and heating at 100°C for 5 min. Plates were analyzed under UV at 366 nm.

### 2.4 Animals and experimental design

**Figure 1:**
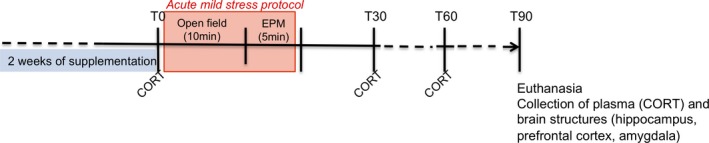
Experimental protocol in adult Balb/c mice

Seven‐week‐old male Balb/c mice, a highly stress‐sensitive strain (Janvier, Le Genest‐Saint‐Isle, France), were housed under a normal 12‐hr light/dark cycle (07 hr–19 hr) with food (AO4 diet; Safe, Augy, France) and water available ad libitum in a controlled environment (22 ± 1°C, 40% of humidity). The mice were handled daily for 1 week before the start of the experiment to minimize stress reactions to manipulation. During the following 2 weeks, they received each morning a supplement comprising either *R. rosea* HRE (a 5 g/kg solution containing 80% glycerin, i.e., 4 g/kg; test group, *n* = 8) or glycerin alone (4 g/kg; control group, *n* = 8) administered by gavage using a V0105040 feeding probe (ECIMED, Boissy‐Saint‐Léger, France). The two groups received the same amount of glycerin. The volume of supplementation was adapted to the weight of each mouse. At the end of this period, the mice were subjected to an acute mild stress protocol and anxiety‐like behavior was evaluated. Blood was drawn from the mandibular vein before initiation of the stress protocol (at t0 min) and then at t30 min and t60 min. Mice were sacrificed at t90 min, and brain structures (hippocampus, hypothalamus, and amygdala) and plasma were excised and frozen at −80°C (Figure 1).

### 2.5 Induction of acute mild stress

On the last day of supplement administration, half the mice in each group were subjected to acute mild stress. The stress protocol consisted in subjecting the mice to an open‐field (OF) test for 10 min immediately followed by an elevated plus maze (EPM) test for 5 min (see the following sections for details; Figure 1). Experiments were performed in the morning, one hour after gavage, under conditions of dim light and low noise. Both tests induce mild stress in animals by subjecting them to anxiogenic conditions (Treit, Menard, & Royan, 1993).

### 2.6 Evaluation of anxiety‐like behavior

Anxiety‐like behavior was evaluated after induction of acute mild stress as previously reported by Dinel et al. (2011). Mouse behavior was videotaped and scored using “Smart” software (Noldus, Wageningen, Netherlands).

#### 2.6.1 OF test

Mice were exposed to an unfamiliar square (40 × 40 cm) OF from which escape was prevented by surrounding walls (16 cm high). The apparatus was virtually divided into 4 central squares defined as the central area (anxiogenic) and 12 squares along the walls, defined as the periphery. Each mouse was placed in the central area and allowed to freely explore the OF for 10 min. Parameters recorded to evaluate anxiety‐like behavior comprised the number of entries into the central area and the percentage of time spent in this area (Dinel et al., 2011).

#### 2.6.2 EPM test

The EPM was a plus‐shaped acryl maze with two opposing open arms (30 × 8 cm) and two opposing closed arms (30 × 8 × 15 cm) connected by a central platform (8 × 8 cm), elevated 120 cm above the floor. Each mouse was placed in the center of the maze facing an open arm, a situation that is highly anxiogenic. The test was performed over a period of 5 min. The number of arm entries and the percent of time spent in open arms were calculated to evaluate the basal level of anxiety. An entry was scored as such only when the mouse placed all its four limbs in any particular arm. A reduction in the percentage time spent in the open arms and the number of entries into these is considered as an index of anxiety‐like behavior, independent of locomotor activity (Dinel et al., 2011).

### 2.7 Biochemical measurements

#### 2.7.1 Measurement of corticosterone

Corticosterone was measured in plasma before and 30, 60, and 90 min after initiation of the stress protocol, using a DetectX corticosterone immunoassay kit (Euromedex, Strasbourg, France) (Dinel, Joffre, et al., 2014).

#### 2.7.2 Assessment of RNA expression using Fluidigm microfluidic arrays

One microgram of total RNA was obtained from each brain area as described in Dinel et al. (Dinel, Andre, et al., 2014) and was reverse‐transcribed with SuperScript III reverse transcriptase (Invitrogen, Cergy‐Pontoise, France). Diluted cDNA (1.3 µl, 5 ng/µl) was added to DNA Binding Dye Sample Loading Reagent (Fluidigm), EvaGreen (Interchim, Montluçon, France), and Tris‐EDTA (TE) buffer with low EDTA to constitute the Sample Mix plate. In the Assay Mix plate, 10 µl of primer pairs (100 µM) was added to the Assay Loading Reagent (Fluidigm) and TE buffer with low EDTA to a final concentration of 5 µM. After priming of the chip in the Integrated Fluidic Circuit Controller, Sample Mix (5 µl) and Assay Mix (5 µl) were loaded into the sample inlet wells. One well was filled with water as a contamination control. To verify specific target amplification and quantitative polymerase chain reaction (Q‐PCR) process efficiencies, a control sample (mouse gDNA, Thermo Fisher, Waltham, USA) was treated, preamplified, and quantified in a control assay (RNasePTaqMan probe, Thermo Fisher) using the same process in the same plate at the same time. The expected value of cycle quantification was around 13. The chip was inserted into the IFC controller, in which 6.3 nl of Sample Mix and 0.7 nl of Assay Mix were blended. Real‐time PCR was performed using the Biomark System (Fluidigm) on the GenoToul platform (Toulouse, France) with the following protocol: Thermal Mix at 50°C, 2 min; 70°C, 30 min; 25°C, 10 min, Uracil‐DNA N‐glycosylase (UNG) at 50°C, 2 min, Hot Start at 95°C, 10 min, PCR Cycle of 35 cycles at 95°C, 15 s; 60°C, 60 s and Melting curves (from 60°C to 95°C). Results were analyzed using the Fluidigm Real‐Time PCR Analysis software v.4.1.3. (San Francisco, USA) to control specific amplification for each primer. Then, the raw data of the qPCR were analyzed using GenEx software (MultiD analyses AB, Freising, Germany) in order to choose the best reference gene for normalizing mRNA expression and to measure the relative expression of each of the 93 genes analyzed in the group receiving the HRE and the control group. GAPDH was found to be the best reference gene in this experiment and was therefore used for normalization of gene expression.

### 2.8 Statistical analysis

#### 2.8.1 Bivariate statistical analysis

All data were expressed as the mean value ± *SEM* (standard error of the mean). A p‐value of 0.05 was considered as significant. Data were analyzed using a one‐way ANOVA (one factor: supplementation) or a two‐way ANOVA with supplementation (HRE, control), and stress (stress; no stress) as between factors followed by a Bonferroni post hoc analysis when interaction was significant (GraphPad software, La Jolla, US). Heatmaps were obtained using the Permut Matrix program (Caraux & Pinloche, 2005).

#### 2.8.2 Principal component analysis (PCA)

PCA was used to assess the gene expression pattern under stress conditions in the group receiving *R. rosea* HRE and the control group. The PCA is a dimension reduction technique that clusters data into principal components (PC) maximizing the variance of the data considered. These PCs are uncorrelated linear combinations of the initial variables which can be interpreted as a pattern. PCA generates factor loadings which reflect the correlation of each variable with the PC and attributes a PC score for each individual. We selected the number of components using the Cattell criterion. Statistical analyses were performed using the XLSTAT program (Addinsoft, Paris, France).

## 3 RESULTS

### 3.1 Phytochemical profile of *R. rosea* HRE

HPTLC analysis showed that *R. rosea* HRE contains salidroside and rosavin (Supplementary data, Fig. S1A). UHPLC‐MS analysis confirmed the presence of these two compounds (peaks 7 and 15) (Fig. S1B and Table S1). Three monoterpene glycosides corresponding to rhodiolosides E, B (or C) and rosiridin (peaks 13 and 24) and several phenylpropane derivatives, including rosarin and rosin, were identified (peaks 15–16 and 18). Five flavonoids were also detected: herbacetin, kaempferol, rhodamine, rhodopsin, and kaempferol‐7‐O‐rhamnoside (peaks 22, 25, 21, 19, and 23, respectively).

### 3.2 *R. rosea* HRE did not impact behavior in acute mild stress protocol

**Figure 2:**
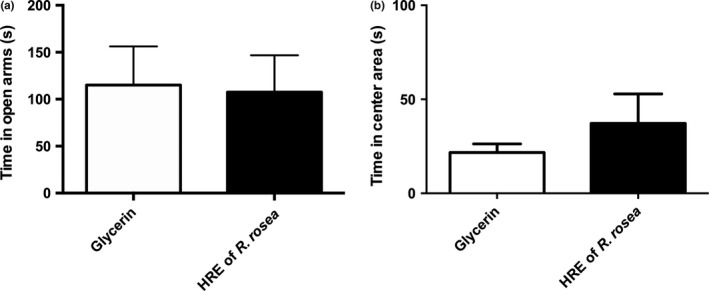
Anxiety‐like behavior of adult mice subjected to acute mild stress having received a *R. rosea* HRE or glycerin (control) supplement for 2 weeks by daily gavage. (a) Time (in seconds) spent in the open arms of the elevated plus maze. (b) Time (in seconds) spent in the center area of the open‐field. Data are presented as means ± *SEM* (*n* = 8 per group). HRE, hydroethanolic root extract

As expected, we did not observed any significant effect of the diet (glycerin or *R. rosea* HRE) on time spent in open arm in the EPM (Figure 2a) or on time spent in center area in the OF (Figure 2b).

### 3.3 *R. rosea* HRE modulated corticosterone secretion consecutive to acute mild stress

**Figure 3:**
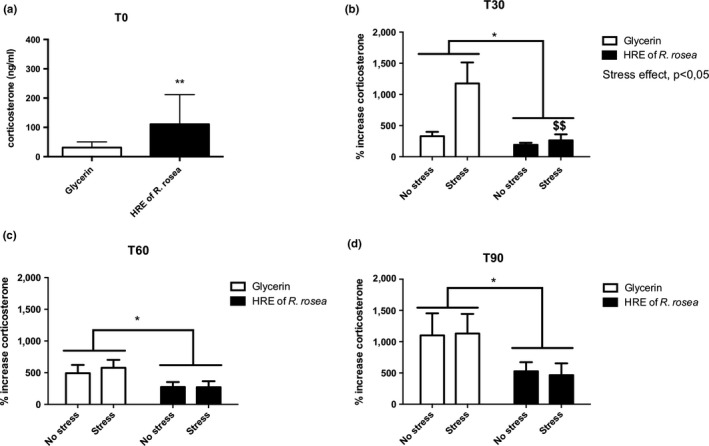
Corticosterone secretion in adult mice having received a *R. rosea* HRE or glycerin (control) supplement for 2 weeks by daily gavage before the induction of acute mild stress (a) and at t30 (b), t60 (c), and t90 min (d) after initiation of the stress protocol. Glycerin versus HRE: *p < .05, **p < .01; glycerin stress versus HRE stress: $$, *p* < .01. HRE, hydroethanolic root extract

Corticosterone was measured in plasma prepared from blood samples drawn before the induction of acute mild stress and 30, 60, and 90 min after the start of the stress protocol. At t0, mice having received *R. rosea* HRE exhibited a significantly higher plasma corticosterone level (110.8 ng/ml) than mice given the control supplement (glycerin alone, 31.31 ng/ml) (*t* = 2.789, *p* < .01; Figure 3a).

A t30, t60, and t90, *R. rosea* HRE induced a decrease in corticosterone secretion compared with the control (*F *(1,24) = 8.352, *p* < .01, Figure 3b; *F *(1,25) = 6.165, *p* < .05, Figure 3c; and *F *(1,26) = 5.954, *p* < .05, Figure 3d, respectively). At t30, we also observed a stress effect (*F *(1,24) = 6.391, *p* < .05, Figure 3b) and a stress × supplementation interaction (*F *(1,24) = 4.544, *p* < .01) indicating that 30 min after the induction of acute mild stress, administration of *R. rosea* HRE restored corticosterone secretion to the basal level.

### 3.4 *R. rosea* HRE modulated stress‐responsive gene expression in a structure‐dependent manner

The expression of 93 genes implicated in stress reactivity was analyzed. Administration of *R. rosea* HRE modulated the pool of stress‐responsive genes described by Datson et al. (2008, 2012), Andrus et al. (2012), and Kohrt et al. (2016). The genes modulated differed between the hippocampus, PFC, and amygdala and could be classified by function. All significant genes and results are presented in Tables 1 and 2.

**Table 1:** Stress‐responsive genes studied by high‐frequency RT‐qPCR in the prefrontal cortex, hippocampus, and amygdala

Symbol	Name	Category	Sequence (5′−3′)	References
TUBB2‐F	Tubulin, beta 2A class IIA	Neuronal structure	TCGGCGCTAAGTTTTGGGAG	Datson et al., EJP 2008
TUBB2‐R			TGCAAGTCACTGTCGCCATG	
NEFL‐F	Neurofilament, light polypeptide	Neuronal structure	TGCAGACATTAGCGCCATGC	Datson et al., EJP 2008
NEFL‐R			TCTCGCTCTTCGTGCTTCTCAG	
GPM6A‐F	Glycoprotein m6a	Neuronal structure	ACTGCTGGAGACACACTGGATG	Datson et al., EJP 2008
GPM6A‐R			AAGAAAGCAGCCGCAATGCC	
LIMK1‐F	LIM domain containing, protein kinase	Neuronal structure	TCCGAGCACATCACCAAAGG	Datson et al., EJP 2008
LIMK1‐R			AGGCGAGGCAGATGAAACAC	
PPP3CA‐F	Protein phosphatase 3, catalytic subunit, alpha isoform	Neuronal structure	CTGGTCGCTGCCATTTGTTG	Datson et al., EJP 2008
PPP3CA‐R			ATCGTCGGAGCAGATGTTGAG	
PFN1 F1	Profilin 1	Neuronal structure	ATCGTAGGCTACAAGGACTCGC	Datson et al., EJP 2008
PFN1 R2			AACCTCAGCTGGCGTAATGC	
DNCIC1‐F	Dynein cytoplasmic 1 intermediate chain 1	Glucocorticoid signaling	AACTTCGTGGTTGGCAGTGAG	Datson et al., EJP 2008
DNCIC1‐R			ACCGATGCCTGCTTTGCTTC	
LIS1‐F	Platelet‐activating factor acetylhydrolase, isoform 1b, subunit 1	Glucocorticoid signaling	GATGTGGGAAGTGCAAACTGG	Datson et al., EJP 2008
LIS1‐R			CTGATTTGGCCGCACCATAC	
KIF5C‐F	Kinesin family member 5C	Glucocorticoid signaling	ATGTAAAGGGGTGCACCGAGAG	Datson et al., EJP 2008
KIF5C‐R			ACGTGTCGGTTTGCTTTGCC	
FKBP1a‐F	FK506‐binding protein 1a	Glucocorticoid signaling	TCCTCTCGGGACAGAAACAAGC	Datson et al., EJP 2008
FKBP1a‐R			AGTTTGGCTCTCTGACCCACAC	
ODC1‐F	Ornithine decarboxylase, structural 1	Glucocorticoid signaling	TCGCCAGAGCACATCCAAAG	Datson et al., Hippocampus 2012
ODC1‐R			TTTTGCCCGTTCCAAGAGAAG	
BHLHB2‐F	Basic helix‐loop‐helix family, member e40	Glucocorticoid signaling	AACGGAGCGAAGACAGCAAG	Datson et al., Hippocampus 2012
BHLHB2‐R			ATCCTTCAGCTGGGCAATGC	
CSNK1A1‐F	Casein kinase 1, alpha 1	Glucocorticoid signaling	CGTCGGTGGAAAATACAAACTGG	Datson et al., Hippocampus 2012
CSNK1A1‐R			TCTCGTACAGCAACTGGGGATG	
SGK1‐F	Serum/glucocorticoid‐regulated kinase 1	Glucocorticoid signaling	CGTCAAAGCCGAGGCTGCTCGAAGC	Arteaga et al., PNAS 2008
SGK1‐R			GGTTTGGCGTGAGGGTTGGAGGAC	
ITPR1‐F	Inositol 1,4,5‐trisphosphate receptor 1	Glucocorticoid signaling	ATCGGCCACCAGTTCCAAAG	Mahfouz et al., PNAS 2016
ITPR1‐R			AGCCAAGTAATGCCCTGTAGCC	
HSD11b1‐F	Hydroxysteroid 11‐beta dehydrogenase 1	Glucocorticoid signaling	GGAAGGTCTCCAGAAGGTAGTGTC	This study
HSD11b1‐R			GAGGCTGCTCCGAGTTCAAG	
SGK1‐F	serum/glucocorticoid‐regulated kinase 1	Glucocorticoid signaling	CGTCAAAGCCGAGGCTGCTCGAAGC	Arteaga et al., PNAS 2008
SGK1‐R			GGTTTGGCGTGAGGGTTGGAGGAC	
MAPK1‐F	Mitogen‐activated protein kinase 1	Glucocorticoid signaling	AGCTAACGTTCTGCACCGTG	Datson et al., EJP 2008
MAPK1‐R			TGATCTGGATCTGCAACACGGG	
PER1‐F	Period circadian clock 1	Circadian rythm	TGTCCTGCTGCGTTGCAAAC	This study
PER1‐R			TTGAGACCTGAACCTGCAGAGG	
MAOA‐F	Monoamine oxidase A	Mood regulation	TGAGGTATCTGCCCTGTGGTTC	Datson et al., EJP 2008
MAOA‐R			CCCCAAGGAGGACCATTATCTG	
SIRT2‐F	Sirtuin 2	Mood regulation	TCCACTGGCCTCTATGCAAACC	This study
SIRT2‐R			TTGGCAAGGGCAAAGAAGGG	
APOE‐F	Apolipoprotein E	Lipid metabolism	TGCGAAGATGAAGGCTCTGTG	This study
APOE‐R			GGTTGGTTGCTTTGCCACTC	
ND2‐F	NADH dehydrogenase 2, mitochondrial	Mitochondria	TTCATAGGGGCATGAGGAGGAC	Hunter et al., PNAS 2016
ND2‐R			GTGAGGGATGGGTTGTAAGGAAG	
ND4L‐F	NADH dehydrogenase 4L, mitochondrial	Mitochondria	CCATACCAATCCCCATCACCA	Hunter et al., PNAS 2016
ND4L‐R			GGACGTAATCTGTTCCGTACGTGT	
ATOX1‐F	Antioxidant 1 copper chaperone	Stress oxydant	ACGAGTTCTCCGTGGACATGAC	This study
ATOX1‐R			TGCAGACCTTCTTGTTGGGC	
GPX1‐F	Glutathione peroxidase 1	Stress oxydant	TCGGACACCAGAATGGCAAG	This study
GPX1‐R			AGGAAGGTAAAGAGCGGGTGAG	

Abbreviation: CORT, dosage of corticosterone.

**Table 2:** Expression of stress‐responsive genes in the hippocampus, prefrontal cortex, and amygdala of adult mice having received a *R. rosea* HRE or glycerin (control) supplement by daily gavage for 2 weeks

Genes	Hippocampus				Prefrontal cortex				Amygdala			
	*p* Value	Stress effect	HRE effect	Interaction effect	*p *Value	Stress effect	HRE effect	Interaction effect	*p* Value	Stress effect	HRE effect	Interaction effect
NEFL	**.00536186**	*	**	ns	**.00359707**	ns	***		.91423389	ns	ns	ns
PPP3CA	**.01171655**	*	*	ns	**.0018328**	ns	***	ns	**.03404985**	ns	*	ns
ND2	**.01182158**	**	ns	ns	.46177831	ns	ns	ns	**.02946051**	ns	ns	*
TUBB2	**.01674524**	ns	**	ns	.13434371	ns	ns	ns	.21949848	ns	ns	ns
PFN1	**.01883924**	*	*	ns	.54380055	ns	ns	ns	.72055469	ns	ns	ns
MAOA	**.01972826**	**	ns	ns	.1495998	ns	ns	ns	.4738274	ns	ns	ns
PER1	**.02003933**	ns	*	ns	**.0044296**	ns	***	ns	**.03513325**	ns	**	ns
SGK1	**.02072644**	*	*	ns	.19595612	ns	ns	ns	.38542471	ns	ns	ns
ATOX1	**.02130576**	ns	*	ns	.27908873	ns	ns	ns	.14358429	ns	ns	ns
DNCIC1	**.02562802**	*	*	ns	.23670027	ns	ns	ns	.63824503	ns	ns	ns
SIRT2	**.02808779**	ns	*	ns	**.04405975**	ns	**	ns	.56556049	ns	ns	ns
LIS1	**.03032655**	ns	*	ns	**.01045478**	ns	**	ns	.30668563	ns	ns	ns
ND4L	**.03105162**	**	ns	ns	.81456675	ns	ns	ns	.38397615	ns	ns	ns
APOE	**.03648538**	*	*	ns	.77427505	ns	ns	ns	.37674375	ns	ns	ns
HSD11b	**.04383912**	*	ns	ns	.63171984	ns	ns	ns	.27091781	ns	ns	ns
FKBP1a	**.04545025**	*	ns	ns	**.00167245**	**	*	ns	.77082307	ns	ns	ns
CSNK2A1	**.04576679**	ns	*	ns	.53585568	ns	ns	ns	.31993563	ns	ns	ns
MAPK1	**.0491574**	ns	*	ns	.05229855	ns	ns	ns	.23113638	ns	ns	ns
LIMK1	.17390743	ns	ns	ns	**.00620715**	ns	**	ns	.47758475	ns	ns	ns
KIF5C	.15161662	ns	ns	ns	**.01669629**	ns	*	ns	**.04889517**	ns	*	ns
GPM6A	.08636875	ns	ns	ns	**.02467013**	ns	**	ns	.13296	ns	ns	ns
BHLHB2	.58012832	ns	ns	ns	**.03217171**	ns	*	ns	.86942235	ns	ns	ns
GPX1	.12956043	ns	ns	ns	**.0422302**	ns	**	ns	.61110372	ns	ns	ns
ODC1	.48378801	ns	ns	ns	.46619784	ns	ns	ns	**.03855515**	*	ns	ns
ITPR1	.48607125	ns	ns	ns	.26712319	ns	ns	ns	**.04987621**	ns	ns	*

Abbreviation: HRE, hydroethanolic root extract.

*** *p* < .001;

** *p* < .01;

* *p* < .05, ns, not significant.

In the hippocampus, 13 genes were significantly overexpressed after repeated administration of *R. rosea* HRE. These genes were implicated in signal transduction (CSNK2A1, *F *(1,22) = 4.694, *p* < .05; MAPK1, *F *(1,22) = 5.248, *p* < .05; SGK1, *F *(1,22) = 6.591, *p* < .05), neuronal structure (NEFL, *F *(1,22) = 8.870, *p* < .01; TUBB2, *F *(1,22) = 8.077, *p* < .01; PPP3CA, *F *(1,22) = 4.396, *p* < .05; PFN1, *F *(1,22) = 4.892, *p* < .05), oxidative stress (ATOX1, *F *(1,22) = 7.753, *p* < .05; APOE, *F *(1,22) = 4.450, *p* < .05, SIRT2, *F *(1,22) = 7.711, *p* < .05) and regulation of the HPA axis (LIS1, *F *(1,22) = 5.623, *p* < .05; DNCIC1, *F *(1,22) = 4.493, *p* < .05). PER1 expression, implicated in circadian rhythm, was also increased after HRE administration (*F *(1,22) = 7.774, *p* < .05). Stress affected the expression of 11 genes including NEFL, *F *(1,22) = 7.624, *p* < .05; PPP3CA, *F *(1,22) = 7.701, *p* < .05; PFN1 *F *(1,22) = 7.359, *p* < .05; SGK1, *F *(1,22) = 5.088, *p* < .05; DNCIC1, *F *(1,22) = 6.041, *p* < .05 and APOE, *F *(1,22) = 4.866, *p* < .05) that were also regulated by *R. rosea* HRE. The mitochondrial genes ND2 (*F *(1,22) = 12.17, *p* < .01) and ND4L (*F *(1,22) = 10.17, *p* < .01) were also upregulated by stress along with MAOA (*F *(1,22) = 10.68, *p* < .01), HSD11b (*F *(1,22) = 7.636, *p* < .05), and FKBP1a (*F *(1,22) = 6.701, *p* < .05), the expression of which is classically induced by chronic or acute stress.

In the PFC, acute mild stress affected only FKBP1a (*F* (1, 16) = 16.10, *p* < .01). *R. rosea* HRE also increased the expression of genes implicated in neuronal structure (NEFL, *F* (1,16) = 16.14, *p* < .001; PP3CA, *F* (1,16) = 19.07, *p* < .001; LIMK1, *F* (1,16) = 14.98, *p* < .01; GPM6A, *F* (1,16) = 8.791, *p* < .01), oxidative stress (SIRT2, *F* (1,16) = 9.914, *p* < .01; and GPX1, *F* (1,16) = 8.822, *p* < .01), HPA axis regulation (LIS1, *F* (1,16) = 12.96, *p* < .01; KIF5C, *F* (1,15) = 6.141, *p* < .05; FKBP1a, *F* (1,16) = 7.889, *p* < .05; BHLHB2, *F* (1,16) = 7.892, *p* < .05), and circadian rhythm (PER1, *F* (1,16) = 16.90, *p* < .001).

The amygdala was less responsive than the hippocampus and PFC to *R. rosea* HRE, only six genes being modulated by this supplement and/or stress. As in the other structures, PPP3CA, KIF5C, and PER1 were overexpressed following *R. rosea* HRE administration (*F *(1,18) = 8.174, *p* < .05; *F *(1,19) = 5.581, *p* < .05 and *F *(1,19) = 10.06, *p* < .01, respectively). Acute mild stress induced an increase in OD1 expression (*F *(1,19) = 5.575, *p* < .05). Interestingly, ND2 and ITPR1 expressions were similarly increased by HRE administration under stress conditions (stress × supplementation interaction *F *(1,19) = 4.399, *p* < .05; *F *(1,18) = 6.837, *p* < .05, respectively).

**Figure 4:**
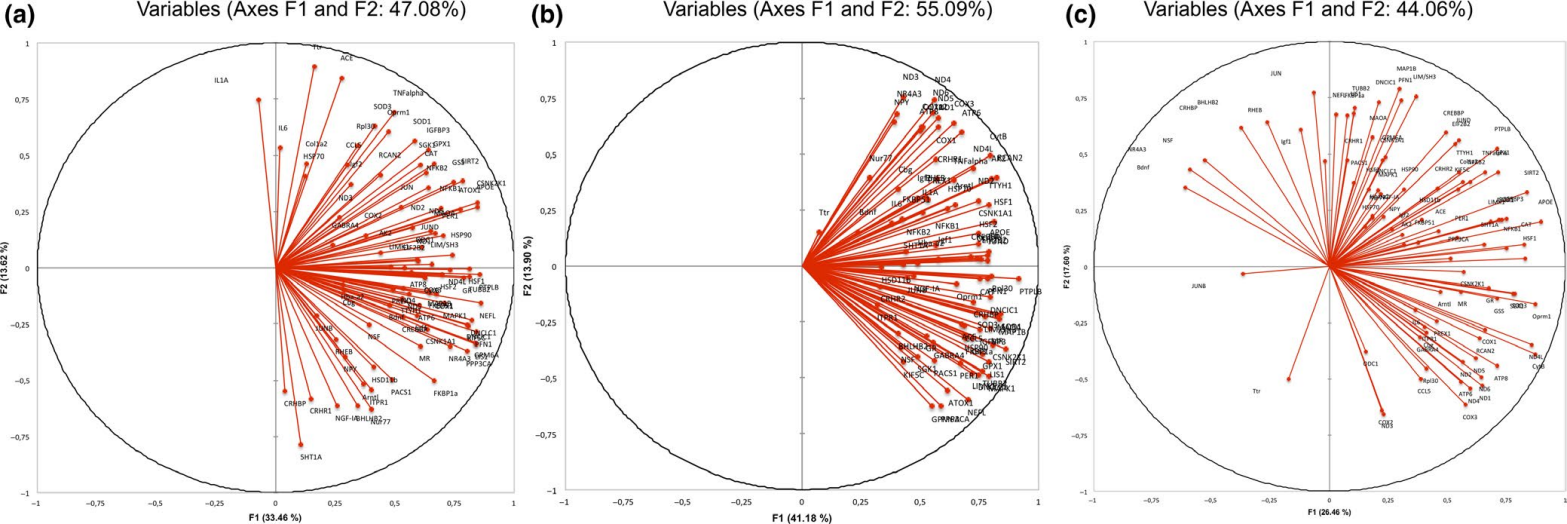
Graphic representation, defined by the first two principal components (F1 and F2), of the Principal Component Analysis (PCA) of gene expression measured by RT‐PCR in the hippocampus (a), prefrontal cortex (b), and amygdala (c) of adult mice having received a *R. rosea* HRE or glycerin (control) supplement by daily gavage for 2 weeks before the induction of acute mild stress. HRE, hydroethanolic root extract

PCA of all genes studied in the hippocampus (Figure 4a), PFC (Figure 4b), and amygdala (Figure 4c) was performed to identify those contributing most to the observed differences between the treatment groups. Remarkably, PCA analysis showed clear separation of the variables: the first component (“F1”) explained 33.46%, 41.18%, and 26.46% of total variance in the hippocampus, PFC, and amygdala, respectively. Pattern 1 revealed that the genes studied were mostly upregulated in the hippocampus and PFC whereas their regulation was more heterogeneous in the amygdala. The second component (“F2”) explained 13.62%, 13.90%, and 17.60% of total variance in the hippocampus, PFC, and amygdala, respectively. This component could reveal a gene classification by functionality.

**Figure 5:**
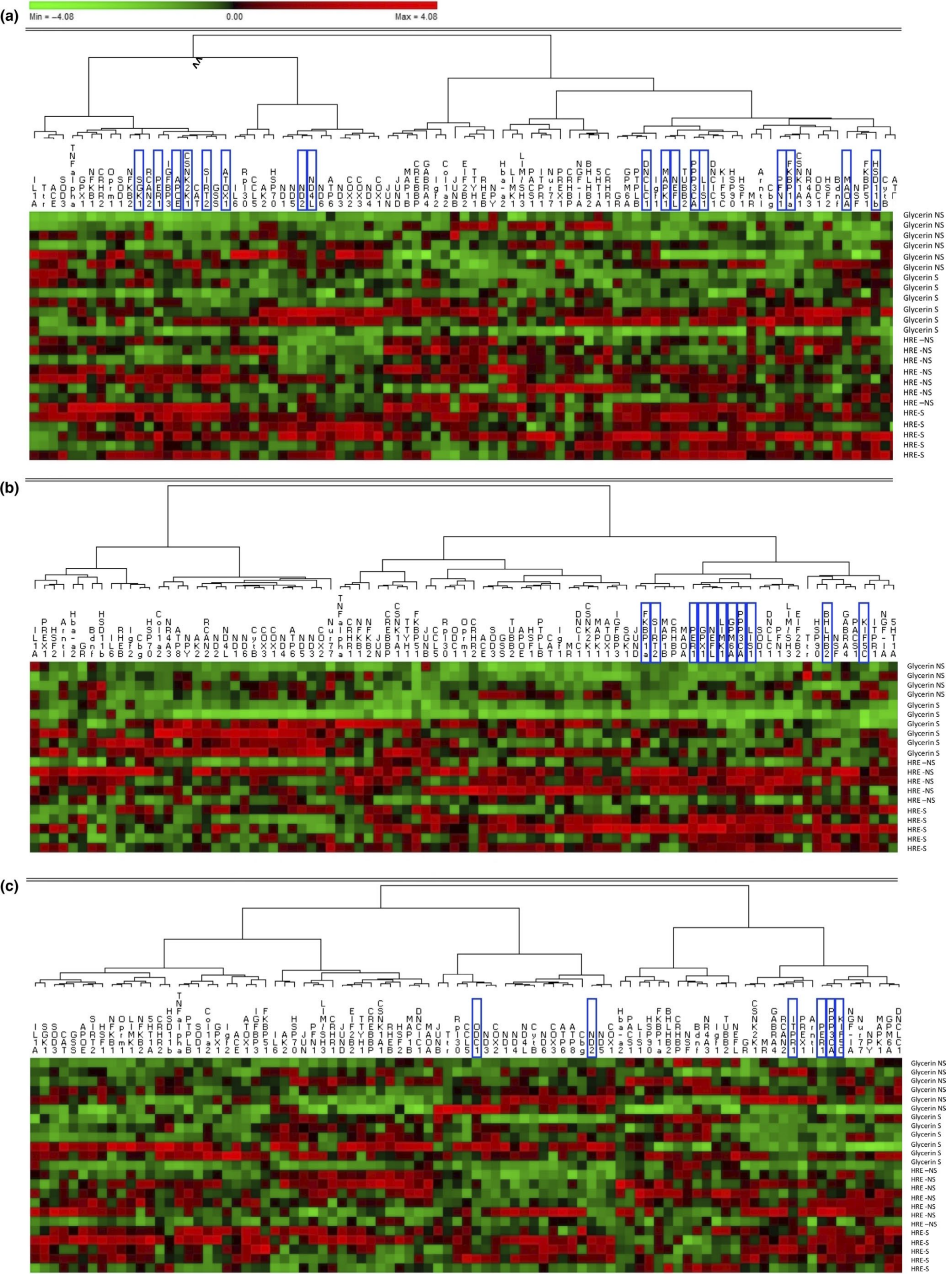
Phylogenic relationship based on Pearson's correlation in the hippocampus (a), prefrontal cortex (b), and amygdala (c) of adult mice having received a *R. rosea* HRE or glycerin (control) supplement for 2 weeks by daily gavage before the induction of acute mild stress. The genes highlighted were modulated by stress, HRE supplementation, or interaction. HRE, hydroethanolic root extract

Phylogenetic analysis based on Pearson's correlation was performed for the three brain structures studied (Figure 5). The heatmap generated demonstrated that gene regulation depends on the group considered (HRE‐supplemented or control), especially as regards the PFC. However, we did not observe any real gene clusters.

## 4 DISCUSSION

The objective of this study was to evaluate the effect on the HPA axis of chronic administration of a *R. rosea* HRE in a murine acute mild stress model by measuring corticosterone secretion and assessing cerebral expression of stress‐responsive genes.

### 4.1 *R. rosea* HRE decreased stress‐induced corticosterone secretion

In the acute mild stress model used in this study, Balb/c mice were consecutively subjected to an OF and an EPM test. We chose to use Balb/c mice as studies have shown this strain to be highly stress‐sensitive compared with other strains (Moloney, Dinan, & Cryan, 2015). Both tests used in this study induce stress in animals by placing them in anxiogenic environments: an open place in the OF test and open arms in the EPM test (Treit et al., 1993).

The basal level of corticosterone was higher in mice receiving *R. rosea* HRE than in control mice receiving a supplement containing glycerin alone. This difference might be explained by the organoleptic characteristics and higher viscosity of the HRE compared with glycerin alone, which could have created additional stress during administration of these supplements (Hoggatt, Hoggatt, Honerlaw, & Pelus, 2010). Even if the percentage of increase was important, the level of corticosterone in mice having received the *R. Rosea* HRE was far below levels obtained after a stress, even in low reactive mice (Mattos et al., 2013). Moreover, we did not observe any behavioral difference in anxiety‐like tests between glycerin‐ and *R. Rosea* HRE‐treated mice.

Thirty minutes after acute mild stress induction, control mice presented, as expected, an increase in corticosterone secretion, whereas mice receiving *R. rosea* HRE did not. At t60 and t90, the percentage corticosterone increase was comparable between stress‐free and stressed mice. We hypothesize that the effect of experimentally induced acute mild stress was masked by that of gavage. We nevertheless observed that at both times, mice having received *R. rosea* HRE presented a lower percentage increase in corticosterone as compared to the control group. This result implies that administration of *R. rosea* HRE resulted in better regulation of stress homeostasis, characterized by more effective control of corticosterone increase that probably led to more efficient restoration of corticosterone level to the basal value.

At the intracellular level, high corticosteroid levels impact the balance between trophic and atrophic factors within neurons (Liu et al., 2017). For instance, glucocorticoids have been shown to inhibit cell proliferation in the dentate gyrus by reducing the proliferation of granule cell precursors (Gould & Tanapat, 1999; Saaltink & Vreugdenhil, 2014). Moreover, chronic stress results in persistent inhibition of granule cell production and changes in the structure of the dentate gyrus, raising the possibility that stress alters hippocampal function through this mechanism (Gould & Tanapat, 1999). By preventing the substantial increase in corticosterone level, *R. rosea* extracts could prevent this negative impact of corticosteroids. Our results confirm those of previous studies demonstrating the impact of *R. rosea* extracts on inhibition of the HPA axis, as illustrated notably by the serum level of corticosteroids in rats (Cifani et al., 2010; Xia, Li, Wang, Wang, & Wang, 2016). The antistress properties of *R. rosea* extracts have been attributed to their interference with both the HPA axis and the sympathoadrenal system (Panossian, Hovhannisyan, et al., 2009; Panossian & Wagner, 2005; Panossian, Wikman, et al., 2009; Panossian, Wikman, & Wagner, 1999). However, all these results were obtained in animals subjected to intense acute or chronic stress. In this study, we demonstrated for the first time that a specific *R. rosea* extract affects HPA axis reactivity even under conditions of mild stress of short duration. The dampening of corticosterone secretion could be due to a decrease in stress reactivity amplitude or to better control of the glucocorticoid pathway.

### 4.2 *R. rosea* HRE upregulated the expression of functional stress‐responsive genes

One of the main mechanisms of action of corticosteroids in the brain is their genomic effect, resulting in modification of target gene transcription. Corticosteroid‐mediated transcriptional changes within the brain have been studied by means of large‐scale gene expression profiling (Datson et al., 2008, 2012; Hunter et al., 2016; Kohrt et al., 2016). The resulting gene expression profile showed a highly dynamic transcriptional response to glucocorticoid receptor activation throughout a specific time window, shifting from exclusively down‐regulation of genes 1 hr after glucocorticoid receptor activation to both up‐ and down‐regulation after 3 hr (Morsink, Steenbergen, et al., 2006). We investigated the impact of *R. rosea* HRE, 1h30 after the induction of acute mild stress, on the expression of stress‐responsive genes (Datson et al., 2008, 2012; Hunter et al., 2016) in the PFC and amygdala, structures involved in the regulation of stress, as well as in the hippocampus, a medial temporal lobe structure implicated in the formation of stable memories and highly susceptible to stress (Kim & Diamond, 2002).

Interestingly, most genes modulated in the PFC, amygdala, and hippocampus by *R. rosea* HRE belong to four main functional groups of genes implicated in the functioning of neuronal structures, glucocorticoid signaling, circadian rhythm, and mood regulation, respectively.

Supplementation with *R. rosea* HRE upregulated genes coding for structural components of the cytoskeleton, such as beta‐tubulin (TUBB2) and neurofilament light polypeptide (NEFL), genes mediating neurite outgrowth, including glycoprotein M6A (GPM6A) (Alfonso, Fernandez, Cooper, Flugge, & Frasch, 2005), as well as genes specifically involved in the dynamics of the actin cytoskeleton of neurons, calcineurin subunit A (PPP3CA), and profilin 1 (PFN1). Genes affecting the actin cytoskeleton were modulated by the HRE in all three brain structures studied, but acute mild stress affected their expression only in the hippocampus. The actin cytoskeleton is involved in the morphology of dendritic spines, and changes in actin cytoskeletal configurations have been postulated to influence long‐term potentiation, affecting synaptic transmission (Meng et al., 2002; Smart & Halpain, 2000). Under stress, these mechanisms are dysregulated and the connectivity between the various brain structures is impaired (Christoffel, Golden, & Russo, 2011). Several studies have demonstrated that stress induces adverse changes in the morphology and strength of hippocampal excitatory synapses, inducing a generalized atrophy of dendrites and spines in the PFC (Goldwater et al., 2009; Sandi et al., 2003; Stewart et al., 2005; Wellman, 2001). By upregulating genes implicated in neuronal structure genes, *R. rosea* HRE might prevent adverse changes in synaptic plasticity and consequently functional disorders, such as those observed in pathological behaviors or depression.

*R. rosea* HRE also had an impact on the glucocorticoid signaling pathway. Glucocorticoids have been shown to modulate motor activity and axonal transport by regulating transcription levels of dynein cytoplasmic 1 intermediate chain 1 accessory subunit polypeptide (DNCIC1), lissencephaly 1 protein (LIS1), and 5c (KIF5C), a member of the kinesin family (Datson, van der Perk, de Kloet, & Vreugdenhil, 2001; Jimenez‐Mateos, Wandosell, Reiner, Avila, & Gonzalez‐Billault, 2005; Kanai et al., 2000; Morsink, Steenbergen, et al., 2006). In our model, *R. rosea* HRE upregulated the expression of DNCIC1, LIS1, and KIF5C in both the PFC and the hippocampus. KIF5C expression was also upregulated in the amygdala, after HRE supplementation. Acute mild stress affected DNCIC1 expression only in the hippocampus. This modulation of gene expression could act as a primer of the glucocorticoid signaling system. In particular, by upregulating these genes, *R. rosea* HRE could modify glucocorticoid receptor trafficking (Harrell et al., 2004), thereby modulating glucocorticoid receptor translocation and consequently glucocorticoid receptor signaling. Our results showed that *R. rosea* HRE modulated glucocorticoid receptor signaling by changing the expression of genes affecting receptor levels and receptor binding affinity preferentially in the PFC and hippocampus. Moreover, FKBP1a, a glucocorticoid receptor cochaperone affecting the binding affinity of ligands to glucocorticoid receptors (Kovacs, Cohen, & Yao, 2005; Kovacs, Murphy, et al., 2005; Riggs et al., 2004; Sakisaka, Meerlo, Matteson, Plutner, & Balch, 2002; Wochnik et al., 2005) was upregulated by acute mild stress in the PFC and hippocampus but its expression was also affected by *R. rosea* HRE in the PFC. In our model, *R. rosea* HRE also induced in the hippocampus an upregulation of CSNK2A1 and MAPK1 expression, two genes involved in glucocorticoid signal transduction. Previous studies showed that acute administration of glucocorticoids downregulates CSNK2A1 (Datson et al., 2001; Morsink, Steenbergen, et al., 2006), but down‐regulation of this gene was not observed under our 90 min postacute mild stress conditions. This increase in hippocampal CSNK2A1 expression under basal and stress conditions in mice receiving *R. rosea* HRE could act as a primer of the system, thwarting the impact of acute mild stress and preventing the negative impact of glucocorticoids.

*R. rosea* HRE could impact circadian rhythm by modulating PER1. Acute exposure to stressors has been shown to increase PER1 expression in hypothalamic nuclei while suppressing PER1 levels in the central nucleus of the amygdala (Al‐Safadi et al., 2014). In this study, we did not observe any impact of acute mild stress on PER1 expression, but *R. rosea* HRE upregulated PER1 in all three brain structures examined. *R. rosea* HRE is therefore likely to have an impact on circadian rhythm. It is important to bear in mind that modulation of PER1 expression could affect the circadian expression of corticosterone itself. Tanaka et al. recently demonstrated that hypertensive rats presented adverse changes in PER1 expression and that this abnormal adrenal circadian clock may affect steroid hormone secretion by the adrenal gland (Tanaka et al., 2019). Nevertheless, we observed this modulation of PER1 at 90 min after stress induction and a supplementary analysis would be necessary to establish a 24 hr time course of gene expression.

Finally, *R. rosea* HRE modulated the expression of SIRT2, a gene implicated in mood regulation. Adverse changes in SIRT2 expression have been reported in mood disorders, with a decrease in SIRT2 expression consecutive to a chronic stress. Treatment with the antidepressant fluoxetine reversed the stress‐induced changes in SIRT2 (Liu et al., 2015). By upregulating SIRT2 expression in the hippocampus and PFC, *R. rosea* HRE could act like an antidepressant. Previous research has demonstrated that salidroside, one of the active substances of *R. rosea* HRE, prevented the development of depression‐like behavior as effectively as fluoxetine (Zhu et al., 2015). The antidepressant effect of *R. rosea* extracts might be mediated by their impact on SIRT2 expression.

Other genes were regulated by *R. rosea* HRE but their modulation depended more strongly on the brain structure considered. In the amygdala, the *R. rosea* HRE and acute mild stress interaction damped the expression of ND2, a mitochondrial membrane respiratory chain gene, suggesting an essential role of mitochondrial activity as an adaptive response to stress, as previously proposed (Vishnyakova et al., 2016). In the PFC, BHLHB2, a gene implicated in neurotrophic factor activity and neuronal excitability, was upregulated by *R. rosea* HRE, suggesting improved communication between neurons.

To conclude, in the model of acute mild stress used, *R. rosea* HRE decreased corticosterone levels and increased the expression of stress‐responsive genes, especially in the hippocampus and PFC. Most of the genes affected are implicated in neuronal structure and could impact synaptic transmission and plasticity as well as the glucocorticoid signaling regulation pathway. This upregulation by *R. rosea* HRE is associated with damping of corticosterone secretion and a faster return to the basal profile. This result could be explained by a greater efficacy of HPA axis feedback with a more appropriate adaptation of the animals receiving *R. rosea* HRE to a new environment. Moreover, *R. rosea* extracts might modulate the circadian rhythm and potentially biological processes driven by the circadian clock. Complementary studies would be needed to reinforce these preliminary data. Mapping of the signaling pathways and transcription factors involved, both in cell cultures and in animal models, could help to decipher the impact of HRE extracts under stress conditions. The new data presented here nevertheless suggest that *R. rosea* HRE could be of value in modulating reactivity to acute mild stress.

## CONFLICT OF INTEREST

This work was funded by Groupe PiLeJe. Financial support was provided to Sigma Clermont for the performance of the chromatographic analyses and to INRA/Nutribrain for the conduct of in vivo experiments (service provision). The specific roles of the authors including Isabelle Guinobert, Claude Blondeau, and Valérie Bardot from Groupe PiLeJe are articulated in the “author contributions” section.

## ETHICAL STATEMENTS

Animal husbandry and experimental procedures were in accordance with the EU Directive 2010/63/EU for animal experiments and were approved by the national ethical committee for the care and use of animals (approval ID A13169).
