# Supplementary figures and images for: Reduction of acute mild stress corticosterone response and changes in stress‐responsive gene expression in male Balb/c mice after repeated administration of a Rhodiola rosea L. root extract
Source: Food Sci Nutr. 2019 Oct 22;7(11):3827–41. doi: 10.1002/fsn3.1249 (PMC6848809; doi:10.1002/fsn3.1249)

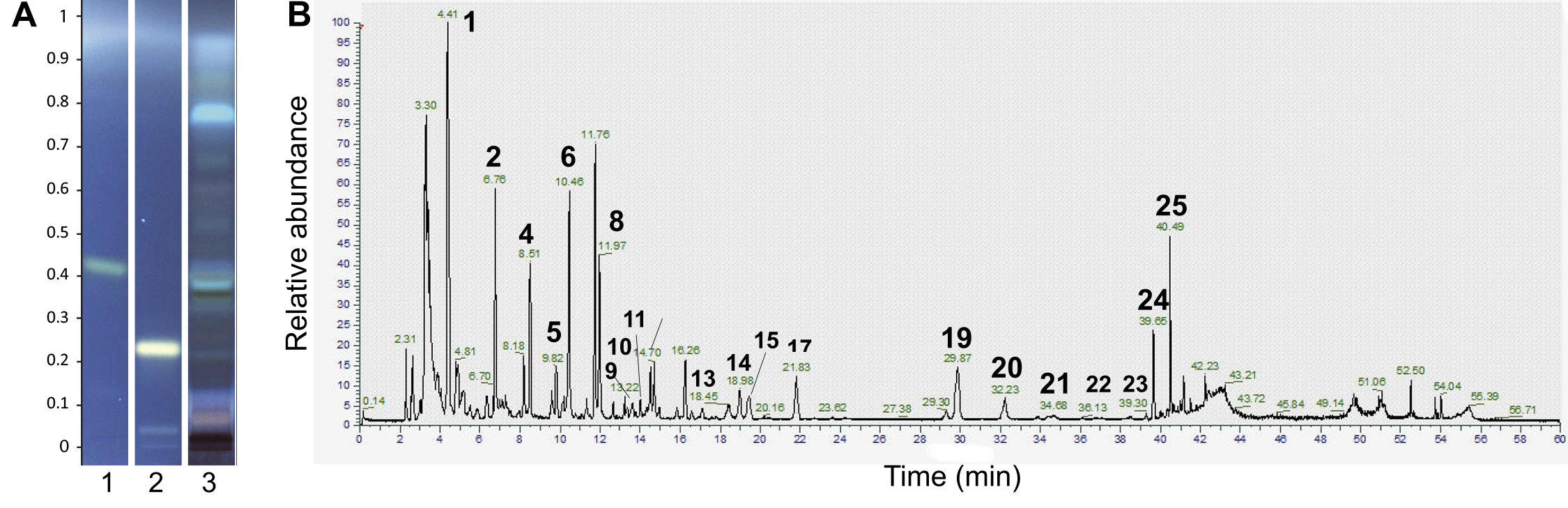

Supplement: Supplementary file 1 [file FSN3-7-3827-s001.tiff]
