# Supplementary material for: Reduction of acute mild stress corticosterone response and changes in stress‐responsive gene expression in male Balb/c mice after repeated administration of a Rhodiola rosea L. root extract
Source: Food Sci Nutr. 2019 Oct 22;7(11):3827–41. doi: 10.1002/fsn3.1249 (PMC6848809; doi:10.1002/fsn3.1249)
Supplement: Supplementary file 3 [file FSN3-7-3827-s003.docx]

**Figure and table captions of supplementary data**:

Figure S1: (A) HPTLC plate showing salidroside (4 µL) in track 1, rosavin (2 µL) in track 2 and *R. rosea* HRE (without glycerin 4 µL) in track 3. (B) Mass spectra of *R. rosea* HRE with attribution of 26 compounds. The compounds corresponding to peaks 1-26 are identified in Table S1. HRE: hydroethanolic root extract.

Table S1: Compounds identified in R. *rosea* HRE in the negative electrospray ionization mode with UHPLC-MS and MS^2^. *: M+[HCOO-]. HRE: hydroetanolic root extract; MS: mass spectrometry; UHPLC-MS: ultra-high-performance liquid chromatography coupled with mass spectrometry.
